# Tensile stress effect on epitaxial BiFeO_3_ thin film grown on KTaO_3_

**DOI:** 10.1038/s41598-018-19487-8

**Published:** 2018-01-17

**Authors:** In-Tae Bae, Tomohiro Ichinose, Myung-Geun Han, Yimei Zhu, Shintaro Yasui, Hiroshi Naganuma

**Affiliations:** 1 Small Scale Systems Integration and Packaging Center, State University of New York at Binghamton, Binghamton, New York 13902 USA; 2 Department of Applied Physics, Graduate School of Engineering, Tohoku University, Sendai, 980-8579 Japan; 3 Condensed Matter Physics and Materials Science, Brookhaven National Laboratory, Upton, NY 11973 USA; 4 Laboratory for Materials and Structures, Tokyo Institute of Technology, 4259-J2-19, Nagatsuda-cho, Midori-ku, Yokohama, 226-8502 Japan; 5 Unité Mixte de Physique, CNRS, Thales, Univ. Paris-Sud, Université Paris-Saclay, 91767 Palaiseau, France

## Abstract

Comprehensive crystal structural study is performed for BiFeO_3_ (BFO) film grown on KTaO_3_ (KTO) substrate using transmission electron microscopy (TEM) and x-ray diffraction (XRD). Nano-beam electron diffraction (NBED) combined with structure factor calculation and high resolution TEM images clearly reveal that the crystal structure within BFO thin film is rhombohedral BFO, i.e., bulk BFO phase. Epitaxial relationship found by NBED indicates the BFO film grows in a manner that minimizes lattice mismatch with KTO. It further suggests BFO film is under slight biaxial tensile stress (~0.35%) along in-plane direction. XRD reveals BFO lattice is under compressive stress (~1.6%), along out-of-plane direction as a result of the biaxial tensile strain applied along in-plane direction. This leads to Poisson’s ratio of ~0.68. In addition, we demonstrate (1) why *hexagonal notation* rather than pseudocubic one is required for accurate BFO phase evaluation and (2) a new XRD method that shows how rhombohedral BFO can readily be identified among other phases by measuring a rhombohedral specific Bragg’s reflection.

## Introduction

BiFeO_3_ (BFO) is known as a multiferroic oxide material with ferroelectricity and *G*-type antiferromagnetism^1,2^. Since its muliferroic properties exist at room temperature, it has been under intense scientific studies for possible applications such as magneto-electric electronics, piezoelectronics, and, especially, low power spintronics technology that makes use of magnetization rather than electrical charge to store information. A number of experimental studies have reported that when BFO is epitaxially grown on single crystal substrates its remarkable physical properties change owing to the lattice strain caused by lattice mismatch (and/or different crystal structure) with the substrates^3^. It was further discussed that crystal structure in BFO film is highly flexible to adapt many percent of lattice strain from substrates which would, otherwise, cause fracture in its bulk form. This leads to experimental findings of various BFO phases such as rhombohedral^4–7^, tetragonal-like^8–12^, orthorhombic^13^, monoclinic^14–18^, and triclinic^19^ depending on crystal structures as well as lattice parameters of substrates used. In addition, theoretical calculations demonstrate existence of multiple metastable BFO phases as a function of strain and temperature^20^. However, because of remarkably complex nature in BFO crystal structure, the number of publications dealing with structural characterization within BFO thin films has not decreased over the recent years as pointed out by a recent review article^21^. This implies the crystallographic details about eptaxially grown BFO thin films remain an open question^21^.

It is worth noting that most of the previous experimental studies have utilized x-ray scattering based techniques making discussions about lattice distortion and/or lattice size changes. While x-ray scattering technique is excellent in providing volume-averaged lattice stress or strain with exceptional precision, the technique does not readily deliver wide range reciprocal lattice information, which is critical to properly evaluate crystal structure in thin film crystals. On the other hand, transmission electron microscopy (TEM) technique readily provides two-dimensional reciprocal space information as wide as *Q* (scattering vector) = 220 nm^−1^ ^22,23^. Furthermore, if TEM technique is combined with SF calculation it has advantage over x-ray scattering technique in that not only lattice distortion and/or lattice size change, but also *locations of each basis atom* in unit cell can be precisely determined. This is particularly important for complex oxide materials such as BFO because slight change in locations of each constituent atom can readily cause extra Bragg’s reflections at unexpected locations in reciprocal space. Recently, we demonstrated the effectiveness of TEM technique combined with structure factor (SF) and first-principles calculations to precisely evaluate crystal structures within epitaxial BFO films grown on various single crystal substrates^6,7,18^.

The objectives of current study are as follows: (1) lattice strain effect in crystal structure of BFO thin film using (100) KTaO_3_ (KTO) substrate which is expected to impart ~0.76% of biaxial tensile stress to BFO (if BFO is assumed as a pseudocubic with *a* = 0.396 nm)^24,25^, (2) growth behavior of BFO utilizing multi zone axes TEM analysis, and (3) demonstration of the existence of a Bragg’s reflection characteristic of rhombohedral BFO using x-ray diffraction (XRD) combined with two dimensional area detector.

## Results and Discussion

**Figure 1:**
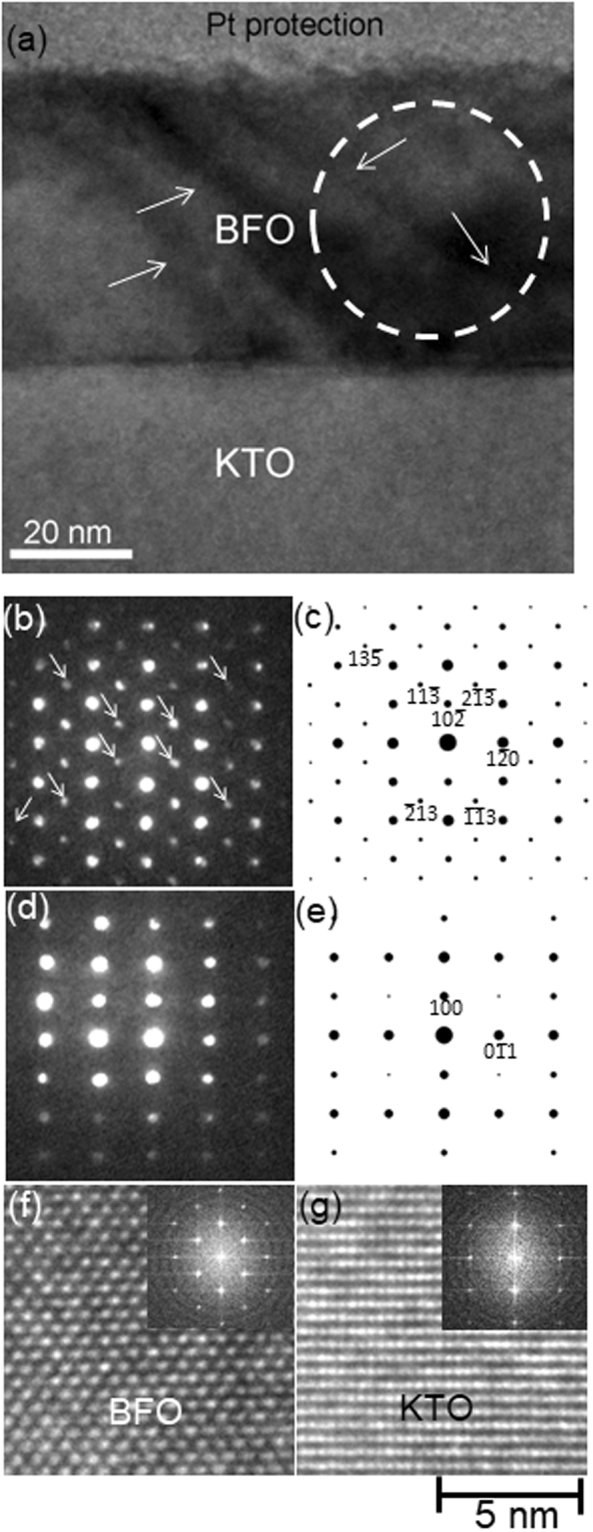
(**a**) Cross sectional BF TEM image of ~60 nm BFO layer along [011]_KTO_ zone axis with NBED patterns from (**b**) BFO and (**d**) KTO substrate. The corresponding structure factor calculations are shown in (**c**) and (**e**), respectively. HRTEM images from BFO and KTO are shown in (**f**) and (**g**) with the corresponding FFT patterns as insets.

Figure 1(a) is a cross-sectional BF TEM image of ~60 nm BFO grown epitaxially on (100) KTO along [011]_KTO_ zone axis. Note that while BFO layer shows lattice strain contrasts as denoted by white arrows it doesn’t show contrasts associated with grain boundary or dislocation. This indicates lattice stress in BFO may not be relaxed. In order to investigate crystal structure within BFO layer, a nano-beam electron diffraction (NBED) pattern was obtained from a BFO area denoted by a white circle with a probe size of ~40 nm as shown in Fig. 1(b). A NBED pattern from an undistorted area within KTO substrate was acquired as shown in Fig. 1(d) to precisely calibrate Bragg’s reflections shown in Fig. 1(b). It is clearly shown that the symmetry and locations of Bragg’s reflections in Fig. 1(b) is different from that in Fig. 1(d), indicating crystal structure of BFO is different from KTO, i.e., cubic perovskite. In an effort to identify BFO crystal structure, lattice spacings as well as the symmetry of the Bragg’s reflections are carefully compared with the calculated structure factor, *F*_*hkl*_, where *hkl* represents a specific Bragg’s reflection. Note that structure factor calculation was performed for all the BFO phases found experimentally as well as metastable ones predicted theoretically, which provide all the necessary crystallographic information including not only lattice parameter but also *locations of basis atoms* in unit cell. These BFO structures include: rhombohedral (space group: *R3c*, *a* = 0.5678 nm, *c* = 1.3982 nm, *α* = *β* = 90°, *γ* = 120°)^26^, monoclinic (space group: *P2*_1_*/m*, *a* = 0.5615 nm, *b* = 0.7973 nm, *c* = 0.5647 nm, *α* = 90°, *β* = 90°, *γ* = 90.1°)^27^, tetragonal (space group: *P4mm*, *a* = 0.367 nm, *c* = 0.464 nm)^20^, monoclinic (space group: *Pc*, *a* = 0.7291 nm, *b* = 0.5291 nm, *c* = 0.5315 nm, *α* = 90°, *β* = 139.46°, *γ* = 90°)^20^, monoclinic (space group: *Cm*, *a* = 0.9354 nm, *b* = 0.7380 nm, *c* = 0.3804 nm, *α* = 90°, *β* = 86.60°, *γ* = 90°)^20^, orthorhombic (space group: *Pna2*_1_, *a* = *b* = 0.5314 nm, *c* = 0.9452 nm, *α* = *β* = *γ* = 90°)^20^, monoclinic (space group: *Cc*, *a* = 1.0604 nm, *b* = 0.5322 nm, *c* = 0.5323 nm, *α* = 90°, *β* = 62.80°, *γ* = 90°)^20^, orthorhombic (space group: *Pnma*, *a* = 0.5650 nm, *b* = 0.7770 nm, *c* = 0.5421 nm, *α* = *β* = *γ* = 90°)^20^, orthorhombic (space group: *Pna2*_1_, *a* = 0.5702 nm, *b* = 0.5507 nm, *c* = 0.8036 nm, *α* = *β* = *γ* = 90°)^20^, and orthorhombic-like monoclinic (space group: *Cm*, *a* = 0.9262 nm, *b* = 0.7582 nm, *c* = 0.3791 nm, *α* = *γ* = 90°, *β* = ~90°)^18^. In addition, structure factor for KTO substrate was calculated as well to investigate its epitaxial relationship with BFO overlayer^28^. The electron diffraction calculation was based on kinematical approximation:

$${F}_{hkl}=\sum _{n}{f}_{n}\exp [2\pi i(h{x}_{n}+k{y}_{n}+l{z}_{n})],$$

where *f*_*n*_ is the atomic scattering factor for atom *n* at fractional coordinates (*x*_*n*_, *y*_*n*_, *z*_*n*_). Details about the electron diffraction pattern analysis and structure factor calculation have been given elsewhere^6^. As a result, it is confirmed that NBED pattern from BFO, i.e., Fig. 1(b) corresponds to [211] zone axis of rhombohedral BFO, i.e., bulk BFO crystal structure [26], as shown in Fig. 1(c). Note that while all of the previous works used pseudocubic notation by *disregarding 0*.*55*° *of rhombohedral distortion* from cubic, we have been using hexagonal notation for BFO to accurately describe its rhombohedral distortion (space group: *R3c*, *a* = 0.396 nm, *α* = 89.45°) from cubic perovskite^26,29^. Bragg’s reflections denoted by white arrows in Fig. 1(b) are due to double diffraction as discussed previously^6,7,18,30^. The NBED pattern from [011]_KTO_ zone axis, i.e. Fig. 1(d) matches its structure factor calculation, i.e., Fig. 1(e), as expected. Based on this analysis, the crystal orientation relation between BFO and KTO is as follows:

[211] of BFO // [011] of KTO; $(10\overline{2})$ of BFO // (100) of KTO (see Fig. 1(c,e)). In order to ensure that extra Bragg’s reflections such as $(11\overline{3})$, $(2\overline{13})$, $(\overline{11}3)$ and $(\overline{2}13)$ appearing in Fig. 1(b) are not associated with CCD camera artifact, high resolution TEM (HRTEM) images from BFO layer as well as KTO substrate are obtained as shown in Fig. 1(f,g), respectively, with their corresponding fast Fourier transform (FFT) patterns (see insets). While Fig. 1(f,g) are acquired from the same HRTEM image, i.e., under the same illumination and defocus conditions, one can readily find the contrasts between Fig. 1(f,g) are different. This indicates different crystal structures between BFO overlayer and KTO substrate. In addition, the aforementioned extra Bragg’s reflections are (see Fig. 1(b)) found only in FFT pattern in Fig. 1(f). This clearly indicates the extra Bragg’s reflections are resulting from BFO crystal structure, i.e., *rhombohedral*. In fact, these extra reflections from NBED and FFT patterns were previously found for pulsed laser deposition grown BFO films grown on LaAlO_3_ substrate, but no detailed discussion was given^31,32^. On the other hand, we have recently demonstrated that those are associated with bulk rhombohedral crystal structure, i.e. for the BFOs grown on SrTiO_3_ and LaAlO_3_ substrates^6,7,18^. The current result turns out consistent with these previous reports^6,7,18^, suggesting slight rhombohedral distortion from cubic perovskite, i.e. *α* = 89.45°, cannot be disregarded for accurate BFO phase analysis.

**Figure 2:**
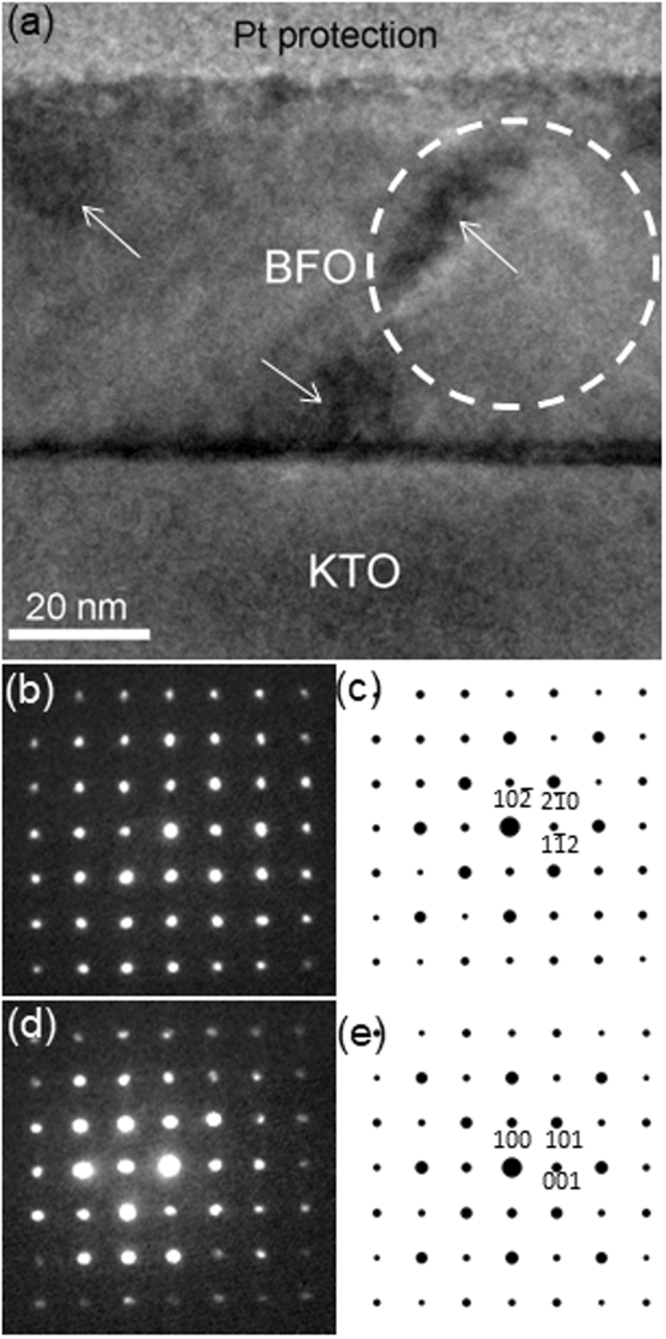
(**a**) Cross-sectional BF TEM image of ~60 nm BFO layer along [010]_KTO_ zone axis with NBED patterns from (**b**) BFO and (**d**) KTO substrate. The corresponding structure factor calculations are shown in (**c**) and (**e**), respectively.

**Figure 3:**
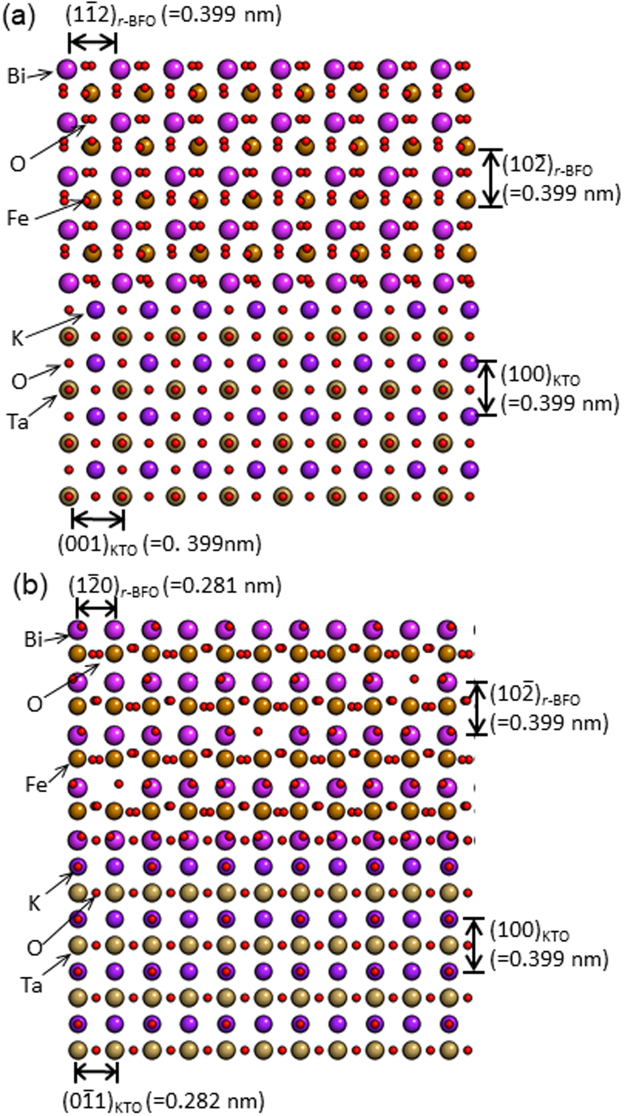
Atomistic models at BFO/KTO interface along (**a**) [241]_BFO_//[010]_KTO_ and (**b**) [211]_BFO_//[011]_KTO_ zone axes.

Since a NBED pattern is a two dimensional cross section out of three dimensional reciprocal space, it is necessary to examine the BFO overlayer from a different orientation to verify the reciprocal space information. Thus, an additional cross sectional TEM sample was prepared along [010] zone axis of KTO substrate, which is 45° away from [011]_KTO_ zone axis, as shown in Fig. 2. A BF TEM image in Fig. 2(a) exhibits ~60 nm BFO epitaxial layer grown on KTO substrate. Note that it shows lattice strain contrasts (as marked by white arrows) with no sign of lattice imperfections, indicating lattice strain in BFO may not be relaxed. This is consistent with Fig. 1(a). The corresponding NBED patterns from BFO and KTO are shown in Fig. 2(b,d), respectively. Note that the NBED pattern from BFO was obtained using ~40 nm probe as denoted by a while circle in Fig. 2(a). Interestingly, both of Fig. 2(b,d) show four fold symmetry. The four fold symmetry in Fig. 2(d) is expected to be related to [010] zone axis of KTO of which crystalline structure is cubic perovskite as proved by the corresponding structure factor calculation in Fig. 2(e). On the other hand, the four fold symmetry in Fig. 2(b) is attributed not to [010] cubic perovskite but to [241] zone axis of rhombohedral crystal structure in BFO as demonstrated by the corresponding structure factor calculation shown in Fig. 2(c). Note that the angle between [241]_BFO_ and [211]_BFO_ is calculated to be ~45° which is the same as that between [010]_KTO_ and [011]_KTO_. This clearly demonstrates the validity the current NBED pattern analysis. Thus, it is concluded that hexagonal notation rather than pseudocubic one should be used to properly investigate crystal structure of epitaxial BFO because (1) pseudocubic notation cannot explain the existence of extra Bragg’s reflections in Fig. 1(b), and (2) the four fold symmetry in Fig. 2(b) could be misinterpreted as a signature of [100] zone axis of cubic perovskite. Based on these results, the crystal orientation relation between BFO and KTO is found:

[241] of BFO // [010] of KTO; $(10\bar{2})$ of BFO // (100) of KTO (see Fig. 2(c,e)). In order to understand BFO’s growth behavior in terms of lattice mismatch with KTO, atomistic models along [010]_KTO_ and [011]_KTO_ zone axes are constructed as shown in Fig. 3. Note that the models are based on undistorted BFO and KTO. One can readily see lattice plane distances between BFO and KTO along surface normal, i.e., out-of-plane, direction as well as along surface parallel, i.e., in-plane, direction are highly similar when viewed along both of [010]_KTO_ and [011]_KTO_ zone axes (see Fig. 3(a,b)). However, it is worth noting that there is lattice mismatch of ~0.001 nm, i.e. ~0.35%, that gives rise to tensile strain in BFO overlayer when projected along [011]_KTO_ zone axis (see Fig. 3(b)). The lattice mismatch of ~0.35% found here turns out less than half of ~0.76% estimated previously^24,25^. Note that the previous reports made two assumptions: (1) BFO as a pseudocubic (pc) crystal with lattice parameter of 0.396 nm by disregarding rhombohedral distortion of 0.55° from cubic unit cell, and (2) BFO grows with the same crystalline orientation as KTO substrate, i.e., (100)_pc_ BFO growth on (100) KTO. The current data shown in Figs 1, 2 and 3 clearly reveal that those assumptions used in the previous reports *do not* apply as BFO crystal *cannot* be accurately interpreted by *cubic notation* as has been discussed here. This indicates: (1) hexagonal notation that includes rhombohedral distortion of 0.55° should be used to adequately describe crystal structure of epitaxial BFO and (2) the epitaxial relationship found using hexagonal notation for BFO reveals the lattice mismatch at BFO/KTO interface is ~0.35%. In fact, the epitaxial relationship found here is the same as the one found previously between BFO and STO substrate in which rhombohedral BFO was grown with ~2.5% compressive strain^6,7^. This suggests that BFO could grow epitaxially by maintaining its bulk rhombohedral crystal structure when its lattice mismatch with substrate falls between ~2.5% compressive and ~0.35% tensile strains.

**Figure 4:**
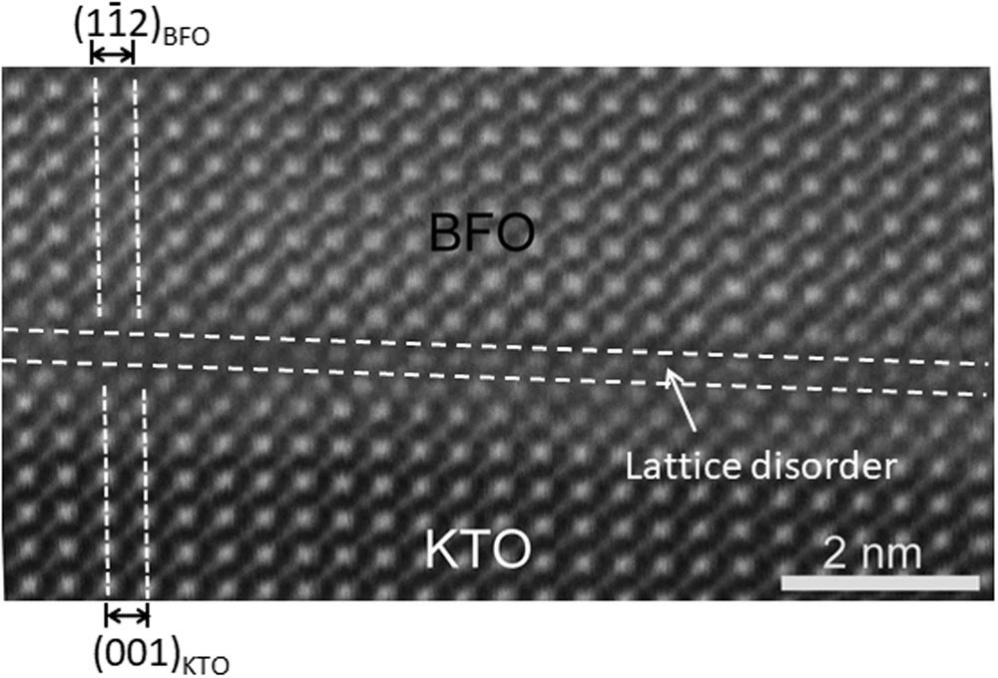
HAADF-STEM image demonstrating lattice planes running smoothly across KTO/BFO interface.

A high angle annular dark field (HAADF)-STEM image was obtained along [010]_KTO_ zone axis as shown in Fig. 4 to directly compare with the atomistic model. Note that the in-plane lattice within KTO (denoted as (001)_KTO_) runs smoothly across the interface toward the in-plane lattice with BFO (denoted as ${(1\overline{1}2)}_{{\rm{BFO}}}$ with no signs of structural relaxation via misfit dislocations except a couple atomic layers of disorder. This is consistent with the atomistic model shown in Fig. 3(a) and further confirms no sign of structural relaxation found in Figs 1(a) and 2(a).

**Figure 5:**
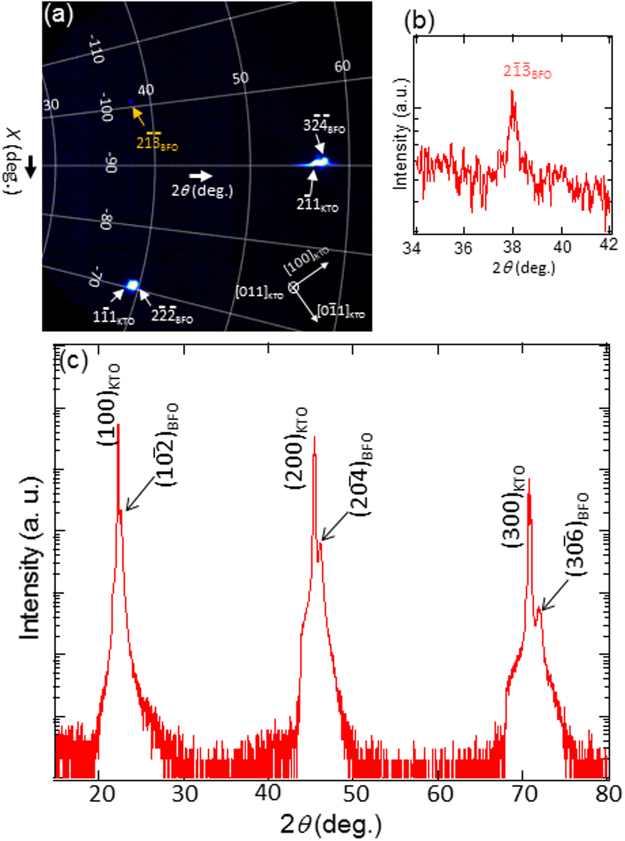
(**a**) X-ray RSM demonstrating the existence of ${(2\overline{13})}_{{\rm{BFO}}}$ Braggs reflection with (**b**) its intensity profile as a function of 2*θ*. (c) XRD with *θ* − 2*θ* geometry, i.e., surface normal direction, demonstrating the locations of ${(10\overline{2})}_{{\rm{BFO}}}$, ${(20\overline{4})}_{{\rm{PFO}}}$, and $(30\overline{6})$ show up at slightly higher 2*θ* than those of (100)_KTO_, (200)_KTO_ and (300)_KTO_.

It is worth pointing out that despite the importance of extra Bragg’s reflections such as $(11\overline{3})$ and $(2\overline{13})$ shown in Fig. 1(b) in terms of BFO crystal structure evaluation, no previous XRD studies have discussed about those reflections. In an effort to provide an evidence of existence of the extra Bragg’s reflections with XRD, x-ray reciprocal space mapping (RSM) is measured around an extra reflection, i.e., ${(2\overline{13})}_{{\rm{BFO}}}$, using an area detector as shown in Fig. 5(a). Note that the intensity of ${(2\overline{13})}_{{\rm{BFO}}}$ shows up quite weaker than other fundamental reflections. This is associated with two factors: (1) the weaker nature of its intensity than BFO fundamental reflections as demonstrated by structure factor calculation (see Fig. 1(c)) and literature^26^, (2) intensity enhancement of BFO fundamental reflections by overlapping with those of KTO substrate. Nonetheless, ${(2\overline{13})}_{{\rm{BFO}}}$ reflection is clearly visible in one dimensional intensity profile as shown in Fig. 5(b). In addition, its angle with ${(3\overline{24})}_{{\rm{BFO}}}$ is measured ~10.1° which is the same as the angle measured between the two reflections in NBED pattern, i.e., Fig. 1(b). This demonstrates the consistency between XRD-RSM, and TEM-NBED results in terms of the existence of the extra Bragg’s reflections that are characteristic of rhombohedral BFO.

It is now clear that (1) BFO overlayer in the current study grows rhombohedral crystal structure and (2) atomistic models as well as atomic-resolution STEM analysis suggest that BFO/KTO interface may be under unrelaxed tensile strain. Thus, it is worth considering how the in-plane tensile stress affects out-of-plane direction. In Fig. 5(c) is shown a XRD result with *θ* − 2*θ* geometry, i.e., scanning along surface normal direction (=out-of-plane), using Cu Kα radiation to obtain volume-averaged data across entire BFO film. As expected from NBED analyses, (100)_KTO_, (200)_KTO_ and (300)_KTO_ peaks show up at 2*θ* = 22.28°, 45.44°, and 70.80° of which lattice spacings correspond to 0.3987 nm, 0.1994nm, and 0.1329 nm, respectively. These values are in excellent agreement with those of undistorted KTO substrate^28^. Given that the lattice spacings of ${(10\overline{2})}_{{\rm{BFO}}}$, ${(20\overline{4})}_{{\rm{BFO}}}$, and ${(30\overline{6})}_{{\rm{BFO}}}$ from undistorted rhombohedral BFO are 0.3994 nm, 0.1997nm, and 0.1331 nm^26^, Bragg’s peaks for those are expected to show up at slightly lower 2*θ* angles than (100)_KTO_, (200)_KTO_ and (300)_KTO_ peaks, respectively. However, the BFO Bragg’s peaks show up at slightly higher 2*θ* angles than each corresponding KTO Bragg’s peaks with their lattice spacings corresponding to 0.3928 nm, 0.1966 nm, and 0.1311 nm, respectively. This clearly indicates BFO lattice spacings along surface normal direction decrease ~1.6%, i.e, compressive stress, as a result of tensile strain applied to BFO along in-plane direction as discussed in Fig. 3. Now, let us think about why compressive strain (~1.6%) along out-of-plane turns out greater than tensile strain (~0.35%) along in-plane. It is worth noting that while in-plane tensile strain is biaxial exerted across the entire area of BFO/KTO interface, i.e., two dimensional, out-of-plane compressive strain is uniaxial exerted along surface normal direction, i.e., one dimensional. Since two dimensional effect, i.e., in-plane strain, should show up as one dimensional effect, i.e., out-of-plane strain, out-of-plane strain (~1.6%) turns out greater than in-plane strain (~0.35%). For this reason, the biaxial stress applied in epitaxial BFO film is reported to yield greater Poisson’s ratio (ν), i.e., ν = ~0.49, than that in BFO film under uniaxial strain, i.e. ν = ~0.30^33,34^. The Poisson’s ratio in the current study turns out ~0.68 by using the equation ν = *ε*_*xx*_/*ε*_*zz*_/(*ε*_*zz*_/*ε*_*xx*_ − 2), where *ε*_*xx*_ and *ε*_*zz*_ are in-plane and out-of-plane lattice mismatches, respectively^33,35^. This value is higher than ~0.49 reported previously^33^. It is worth noting while the sample used in the previous study was grown by pulsed laser deposition that leads to multi-domain structure^33^, our sample is grown by ultra high vacuum sputtering method leading to single domain structure as proved by single domain patterns in NBED and x-ray RSM as well as by single domain microstructure found in TEM and HAADF-STEM images. Since strain can be relaxed at domain boundary, single domain structure found in the current study might help preserve the lattice strain more efficiently than that in the previous study. However, we believe further study is necessary to elucidate this point. Defects or non-stoichiometry contributions to the higher Poisson’s ratio are thought to be minimal because of: (1) highly coherent lattice plane at BFO/KTO interface with no obvious signs of lattice imperfections such as dislocations or domain boundaries (see Figs 1, 2, and 4), and (2) no Bragg’ peaks of secondary phases such as Bi_2_Fe_4_O_9_ and Bi_2_O_3_ showing up in XRD data that are indicative of local non-stoichiometry in BFO film.

### Summary

In summary, crystal structure, growth mechanism, and lattice strain effect within BFO film grown on (100) KTO substrate are investigated using advanced TEM and XRD combined with two dimensional area detector. NBED analysis combined with structure factor calculation unambiguously reveals BFO crystal structure is rhombohedral, i.e., its bulk crystal structure by demonstrating extra Bragg’s reflections characteristic of rhombohedral. This clearly indicates *hexagonal notation* rather than pseudocubic one should be used to properly describe the crystal structure of epitaxially grown BFO film. Furthermore, the analyses found the BFO film grows by maintaining epitaxial relationship that can minimize the lattice mismatch as follows:

[211] of BFO // [011] of KTO; $(10\overline{2})$ of BFO // (100) of KTO,

[241] of BFO // [010] of KTO;$(10\overline{2})$ of BFO // (100) of KTO.

BF TEM and atomic resolution HAADF-STEM images demonstrate BFO/KTO interface is atomistically coherent with no sign of lattice strain relaxation. Atomistic models based on the epitaxial relationship indicate BFO film is under slight tensile stress along in-plane direction. XRD measurement clearly reveals BFO lattice distance decrease ~1.6% along surface normal direction resulting from the biaxial tensile stress along in-plane direction.

## Methods

BFO film was grown epitaxially on a (100) KTO substrate using ultra high vacuum (<2 × 10^−6^ Pa) r.f. magnetron sputtering at 550 °C. Cross sectional TEM samples were prepared using FEI Nova 600 dual beam focused ion beam. Ga ion energy was gradually decreased from 30 to 2 keV with to minimize ion beam induced damage. TEM analysis was performed using (1) JEOL JEM-2100F equipped with Gatan Orius 833 CCD camera specifically designed for precise electron diffraction analysis with electron beam damage resistant scintillator, and (2) JEOL ARM 200 equipped with a probe corrector for atomic resolution scanning TEM (STEM) imaging. For XRD analysis, Bruker D8 discover four circle x-ray diffractometer was used with Cu *Kα* radiation. RSM was recorded using two dimensional area detector (Hi-STAR).
